# One-year trajectory analysis for ADHD symptoms and its associated factors in community-based children and adolescents in Taiwan

**DOI:** 10.1186/s13034-017-0165-4

**Published:** 2017-06-01

**Authors:** Chia-Jui Tsai, Yi-Lung Chen, Hsiang-Yuan Lin, Susan Shur-Fen Gau

**Affiliations:** 1 Department of Psychiatry, Taichung Veterans General Hospital, Taichung, Taiwan; 2 Graduate Institute of Clinical Medicine, College of Medicine, National Taiwan University, Taipei, Taiwan; 3 Department of Psychiatry, National Taiwan University Hospital and College of Medicine, No. 7, Chung-Shan South Road, Taipei, 10002 Taiwan; 4 Graduate Institute of Epidemiology and Preventive Medicine, College of Public Health, National Taiwan University, Taipei, Taiwan

**Keywords:** ADHD, Trajectory analysis, Community sample, Associated factors, Child and adolescent

## Abstract

**Background:**

Several longitudinal studies have shown the partial symptomatic persistence of attention-deficit hyperactivity disorder (ADHD) in clinic-based samples. However, little is known about the patterns and trajectories of ADHD symptoms in community-based populations.

**Methods:**

To differentiate developmental trajectories of ADHD symptoms over 1 year, with a four-wave quarterly follow-up in children and adolescents in the community of Taiwan, we conducted this prospective study in 1281 students in grade 3, 5, and 8. All the students in the regular classes rather than special educational classes were eligible and recruited to the study. Inattention, hyperactivity–impulsivity, and opposition-defiance were rated by parent reports on the Chinese version of the Swanson, Nolan, and Pelham Version IV Scale (SNAP-IV). Group-based trajectory modeling and multivariable regression analyses were used to explore the individual, family and social factors associated with differential trajectories.

**Results:**

Trajectories were classified as Low (29.9–40.6%), Intermediate (52.5–58.5%) and High (6.9–12.5%) based on the symptom severity of ADHD symptoms assessed by the SNAP-IV. The proportion of children in the high ADHD trajectory might approximately reflect the prevalence of ADHD in Taiwan. The following factors differentiated High from Low trajectories: male gender, more externalizing problems, fewer prosocial behaviors, school dysfunction, more home behavioral problems, and less perceived family support.

**Conclusions:**

Our findings that the concurrent conditions of emotional or externalizing problems, as well as impaired school and home function at baseline, might differentiate the high ADHD symptoms trajectory from others could help developing the specific measures for managing high ADHD symptoms over time in a school setting.

**Electronic supplementary material:**

The online version of this article (doi:10.1186/s13034-017-0165-4) contains supplementary material, which is available to authorized users.

## Background

Attention-deficit/hyperactivity disorder (ADHD), characterized by developmentally inappropriate symptoms of inattention, hyperactivity, and impulsivity, is a common childhood-onset neurodevelopmental disorder, with a worldwide-pooled prevalence of 5.29% [1] and 7.5% in Taiwan [2]. Childhood ADHD symptoms onset as early as 4 years of age and adversely affect many functional domains, including unsatisfactory parent–child relationships, poorer academic performance, increased school dropout [3], social dysfunction [4], increased delinquent behaviors and substance use in adolescence [5], alongside unemployment in adulthood [6]. ADHD is mostly diagnosed between 7 and 12 years of age and the persistence or remission of ADHD symptoms, which were highly dependent on the definition of remission used, happened mostly during mid to late adolescent years [7]. However, the understanding of ADHD symptoms trajectories came mainly from clinic-based studies but not from community studies [7, 8]. Identifying the patterns and trajectories of ADHD symptoms in the non-clinical sample has important implications for the guidance and development of effective prevention and management.

Characterizing the persistence of ADHD symptoms is methodologically challenging, partly owing to the complexity in acquiring prospective longitudinal data, provided by a limited number of studies [7–10], several of which relied on clinic-based samples [7, 8]. A meta-analysis has revealed that 15% of adults with a childhood diagnosis of ADHD met full DSM-IV criteria for the disorder at age 25 years, while about 65% were in partial remission [8]. In an 11-year follow-up longitudinal study of boys with ADHD, Biederman et al. found that 35% of children with ADHD continued to meet the full-threshold diagnosis of ADHD, while 43% had partial functional persistence, i.e., they had fewer symptoms than are required for a full diagnosis but remained functionally impaired [7]. In a longitudinal community-based study over a 6-year period, the prevalence of IA symptoms remained stable from early childhood through late adolescence whereas the prevalence of HI symptoms decreased by more than half over time [9]. Although it is easier to recruit participants in clinic, results may be confounded by selection bias, which leads to questionable generalizability to a broader community of interest. Specifically, individuals who show potential ADHD cases but do not have access to health care [11], show low levels of impairment [12], or do not have comorbid psychiatric conditions are less likely to be included in clinical samples than their counterparts. Research about the different persistence patterns of ADHD symptoms in community samples may complement findings from the clinic-based literature.

Investigating the trajectories of ADHD symptoms and their influencing factors may provide insight for the guidance and customization of optimal interventions across developmental stages. However, only a few studies have explored different trajectories of ADHD symptoms and identified associated factors in community samples of children and adolescents. The numbers and trends of trajectories found across studies were inconsistent. For example, Nagin and Tremblay found four levels of trajectory (chronic high, high, moderate, and no problems), in which less than 6% of 1037 boys aged 6–15 years in low socioeconomic areas of Canada were classified as being chronic high trajectory. Who started off scoring high continued to score high throughout the observation period in the hyperactive externalizing behavior section evaluated by the Social Behavior Questionnaire [13]. In a birth cohort of 2593 families in the community, three trajectories with low (78.3–83.3%), moderate (13.4–18.8%), and high (2.8–3.2%) overall symptom levels over time assessed by the ADHD Symptom Checklist were detected in each outcome group [inattention (IA), hyperactive-impulsivity (HI), and total symptoms] [14]. By contrast, several studies only differentiated high- and low-level trajectories for IA and HI symptoms in children [10, 15, 16]. In a community sample of 335 children from high-risk families with alcohol use disorders, those children in the high level of IA/HI severity trajectory rated by subscales of the Child Behavior Checklist had symptoms constantly remained high throughout the course [15]. In a 1450 twin pairs population-based, longitudinal study which developmental trajectories were defined using parent ratings of ADHD symptoms via a checklist of 14 DSM-IV-based items, 14% were included in the high increasing trajectory of IA domain and 9% were included in the high decreasing trajectory of HI domain [16]. Furthermore, the pattern of trajectories also differed across studies; specifically, certain studies reported that HI symptom trajectories decline over time, while IA trajectories remain grossly stable [10]. However, other studies did not support this result. IA trajectories were found to have high increasing or high decreasing trajectories [16, 17]. Also, symptom trajectories might be influenced by the informants. For example, Musser et al. reported that parent-rated HI yielded a 4-class trajectory solution in a latent-class growth analysis (high persistent, high decreasing, moderate decreasing, low decreasing); whereas, teacher-rated symptoms of IA and oppositional defiant disorder (ODD) both yielded a 3-trajectory solution (high persistent, high decreasing, low decreasing in IA, and high worsening, high decreasing, low in ODD) [17]. Several risk factors have been reported to associate with high trajectories of HI and IA subtypes, including large family size, parental divorce, low socioeconomic status, externalizing and internalizing problems [14, 16], parental criticism [17], insufficient parental emotional support, and deficient intellectual stimulation from during early childhood [15]. In contrast to HI and IA symptoms, there is few literature regarding the trajectory and correlates of opposition-defiance (OD) symptoms. OD symptoms, which are highly associated with ADHD, have demonstrated a negative impact on social functioning and ADHD-related behaviors [18]. Hence, it is imperative to differentiate the pattern and trajectory of ADHD core symptoms from OD symptoms.

Given that most ADHD studies focused on clinical rather than non-clinical samples, community-based studies using the trajectory analyses revealed inconsistent results about the patterns and predictors of trajectory. Also, very limited studies have examined the trajectory of OD symptoms. We did not know how these symptoms would change from time to time in a community sample nor did we know its associated factors. The objective of this study was thus to trace the distinct 1-year trajectories of IA, HI, and OD symptoms and to identify the associated factors for these trajectories in a large community sample of Taiwanese children and adolescents. Family function, parenting styles, social and school adjustment, and behavioral problems of participants were thoroughly assessed and tested for their associations with the trajectories of ADHD symptoms. Moreover, in light of previous studies demonstrating that the number of trajectories varied across studies using global ratings for ADHD, we expected to identify between two and four trajectories of ADHD symptoms as the majority literature found. We anticipated to identify at least one trajectory lied in high symptom severity for each symptom domain regardless of their pattern (e.g., increasing, decreasing, flat). We also hypothesized that those belong to the High trajectory would be associated with higher co-occurring externalizing problems, lower function at school and home, and lower perceived family function comparing to those belong the Low and Intermediate trajectories, for high symptom severity samples who get higher total scores on IA or HI domains might mimic clinical ADHD patients. The second objective was to compare cross-sectional differences in the severity of ADHD and OD symptoms across school grades, given that limited studies had investigated symptomatic differences across developmental periods. Declined IA, HI and OD severity with time was observed in a previous community study, especially in those showed high symptom severity [14]. We investigated the severity and trends of the three symptoms related to ADHD to see if they have distinct pattern across age groups (i.e., third graders, fifth graders, and eighth graders).

## Methods

### Subjects and design

This prospective longitudinal questionnaire-based study was conducted using a school-based sample of 1281 students in grade 3, 5, and 8 from Northern Taiwan with a four-wave quarterly follow-up over 1 year of study completion (between February 2013 and January 2014). All the students in the regular classes rather than special educational classes were eligible and recruited to the study. We did not exclude any students with mental disorders in regular classes nor did we include students from special education classes (IQ < 55, in general, as moderate mental retardation or worse). All the students who completed the informed consent were recruited. There were 638 boys and 615 girls at wave 1 (n = 1253); follow-up rates were 93.1% (n = 1166 with 593 boys, 50.5%, and 573 girls, 49.5%), 89.6% (n = 1123 with 563 boys, 48.9%, and 560 girls 51%), and 84.1% (n = 1054 with 563 boys, 48.3%, and 535 girls, 51.7%) at the second, third, and fourth waves, respectively. The numbers of parents who participated in the first four waves were 1128, 1005 (follow-up rate 89.1%), 941 (83.4%), and 849 (75.3%), respectively. The numbers of parents who participated in the first, second, third and fourth waves were 1128, 1005 (follow-up rate 89.1%), 941 (83.4%), and 849 (75.3%), respectively. A portion of the data has been analyzed and published elsewhere [19]. Third- and fifth-grade students were recruited from six elementary schools, and eighth-grade students were recruited from one junior high school. In the current study, grade 3, 5, and 8 represent three developmental periods: childhood, pre-adolescence, and young adolescence.

### Measures

#### ADHD-related symptoms: Chinese version of the Swanson, Nolan, and Pelham Version IV Scale (SNAP-IV)

SNAP-IV is a 26-item rating instrument which includes the core DSM-IV-derived ADHD subscales of IA, HI, and OD (items 1–9, 10–18, and 19–26, respectively) [20]. Each item is rated on a 4-point Likert scale, (0 = “not at all,” 1 = “just a little,” 2 = “quite a lot,” and 3 = “very much,” respectively. Gau et al. [21, 22] have established the norms and psychometric properties of the Chinese version of the SNAP-IV, which demonstrates good test–retest reliability, high internal consistency, and discriminative validity. This questionnaire has been widely used in clinical evaluation and research in Taiwanese child and adolescent populations [23–25]. We used the parent form of the Chinese version SNAP-IV to evaluate ADHD-related symptoms in participants.

#### Externalizing and internalizing behaviors: Chinese version of the Strengths and Difficulties Questionnaires (SDQ)

The SDQ, a 25-item behavioral screening questionnaire, is a brief behavioral screening questionnaire designed to assess the broader psychological problems experienced by children and adolescents. Each behavioral item is rated on a 3-point Likert scale (0 = not true, 1 = somewhat true, and 2 = certainly true) [26]. It has shown good test–retest reliability and moderate to high internal consistency in Taiwan [27]. In this study, we evaluated the prosocial, oppositional-conduct, hyperactivity–inattention, peer problems, and emotional problems based on youth participants’ reports on these subscales of the Chinese version of the SDQ.

#### Family support: the family adaptability, partnership, growth, affection, and resolve (APGAR)

The family APGAR, which consists of five parameters of family functioning: adaptability, partnership, growth, affection, and resolve, is used to assess perceived family support by examining his/her satisfaction with family relationships. Each parameter is assessed by reported satisfaction on a 3-point scale ranging from 0 (hardly ever) to 2 (almost always), with higher scores indicating a better satisfaction and a more highly functional family [28]. The Chinese family APGAR has proved to be a reliable and valid instrument for assessing perceived family support for individuals with mental problems in Taiwan [29–32]. Parent report of the family APGAR was used to assess perceived family supports in the current study.

#### Social and school adjustment: the social adjustment inventory for children and adolescents (SAICA)

The SAICA, a 77-item semi-structured interview scale, provides an evaluation of children’s social adjustment functioning in school, in spare time activities, and with peers, siblings, and parents. It can be administered to school-aged children (aged 6–18) (self-report), or to their parents (who respond regarding their children). A higher mean score indicates either poorer social function or more severe social problems [33]. The Chinese version of the SAICA has been proved to be a reliable and valid instrument for assessing social adjustment across domains in Taiwanese child and adolescent populations [34, 35]. The subscale of school social problems was used to assess children’s behavioral problems at school (e.g., disruptive behaviors, getting into fights, withdrawal, and vandalism) [25, 36]. Students’ behavioral problems at home were assessed by the home behaviors subscale [36]. We used the parent report on the SAICA for the final analysis.

### Procedure

This study was approved by the Research Ethics Committee of the National Taiwan University Hospital (IRB number: 201212010RINC). Informed consent was obtained from all the participants and their parents after the researcher had explained the study purpose, procedures and assured the confidentiality of this study. The participants were collected in a convenience sample of primary and junior high students according to the positive response and cooperation of their school principals. The parents were invited to attend the speech delivered by the corresponding author (SSG) explaining the purpose and procedure of this study. The parents received the informed consent in paper format from their children. Parents who agreed to participate were asked to complete the questionnaire at home and return it in a sealed envelope within 1 week. The students then completed the questionnaires during class under the supervision of research assistants and their teachers. We collected data from participating students and their parents (75% from the mother) in four waves of surveys quarterly within 1 year. The student participants reported on the Chinese SDQ at the first wave. The parents reported on the Chinese versions of the family APGAR, and SAICA about the student participants at the first wave and the Chinese SNAP-IV about the student participants for all four waves of evaluations.

### Statistical analysis

Results are displayed as demographics (frequency and percentage), and as mean and standard deviation (SD) for continuous variables, including the SNAP-IV subscales, SDQ, SAICA, and family APGAR. To address missing data, we conducted the Expected-Maximization algorithm to impute missing variables based on gender, grade, and values from all other available waves.

#### Identification of trajectories

Group-based trajectory modeling analyses were conducted using Proc Traj, a SAS procedure for group-based modeling of longitudinal data [37]. Possible trajectories across four waves for three ADHD dimensions: IA, HI, and OD symptoms were explored using SNAP-IV. The number of trajectories was chosen according to Nagin’s suggestions [38] based on model fit indices, including Bayesian information criterion (BIC) and Akaike information criterion with the possible rational polynomial curve (intercept to cubic). Best fit models with the smallest negative BIC values and change in BIC between two models were considered a measure of evidence for model selection of number and shape of trajectories. If the statistical approach could not be implemented to find the best model, in which the model fit indices continuously decreased when the number of trajectories increased and no inflection point was found, we referred to existing literature to identify the most appropriate number of groups.

#### Correlates of trajectories in each grade

After the number of trajectories had been chosen, subgroup analyzes of group-based trajectory modeling analyses were conducted for each school grade. Multinomial logistic regression analyses carried out with trajectories as outcome and demographics, externalizing and internalizing behaviors, family support, and social and school adjustment as independent variables to identify correlates which could differentiate the trajectories. To further select the independent correlates, we used the stepwise model selection, which tests the addition and deletion of each variable, using p value less than 0.05 as the selection criterion. We used bidirectional elimination approach to conduct the stepwise selection including gender, grade, first wave scores from the SDQ, family APGAR, and family and home function in the SAICA in the initial model.

## Results

Table 1 presents the demographic characteristics, means and SD of the subscales of the Chinese versions of the SDQ, Family APGAR, and SAICA in the first wave, as well as their ADHD-related symptoms in each of the four waves. One-fifth of participants entered the study while they were in grade 3, one-fifth were in grade 5, and 57% of participants were in grade 8. Significant differences in gender and age were identified between the respondents and those excluded (p < 0.05), with fewer dropouts in girls than boys, and fewer dropouts in grades 5 and 3 than grade 8. There were no significant differences in parents’ education level and occupation between dropouts and non-dropouts (p > 0.05). The BIC fit index is shown in Additional file 1: Table S1. Because no best fit model could be found according to the BIC index, we chose three parameters among the three symptom domains, based on the parsimony principle and current literature.

**Table 1:** Sample characteristic and ADHD-related symptoms among each wave

Variables/student report	Wave 1	Wave 2	Wave 3	Wave 4
	Total N = 1281			
Gender, n (%)^a^				
Male	638 (50.9)	589 (50.5)	549 (48.9)	509 (48.3)
Female	615 (49.1)	577 (49.5)	573 (51)	545 (51.7)
Grade, n (%)				
Grade 3	254 (20.3)	219 (18.8)	207 (18.4)	212 (20.1)
Grade 5	281 (22.4)	273 (23.4)	270 (24)	249 (23.6)
Grade 8	718 (57.3)	674 (57.8)	646 (57.5)	593 (56.3)
SDQ, mean (SD)				
Conduct problems	2.04 (1.19)			
Hyperactivity	3.71 (1.35)			
Emotional symptoms	2.00 (1.92)			
Peer problems	4.43 (1.32)			
Prosocial	7.45 (2.07)			
Family APGAR total score, mean (SD)	7.04 (2.97)			
SAICA, mean (SD)				
School function	13.95 (4.02)			
Home behaviors	22.04 (6.60)			
ADHD-related symptoms (SNAP-IV), mean (SD)				
IA	6.90 (4.8)	6.41 (4.4)	6.11 (4.19)	6.19 (4.23)
HI	3.58 (3.96)	3.36 (3.63)	3.10 (3.30)	3.17 (3.54)
OD	4.8 (4.11)	4.57 (3.76)	4.2 (3.76)	4.01 (3.61)

*ADHD* attention-deficit/hyperactivity disorder, *IA* inattention, *HI* hyperactivity–impulsivity, *OD* oppositional-defiance, *SDQ* Strengths and Difficulties Questionnaire, *Family APGAR* family adaptation, partnership, growth, affection, and resolve, *SAICA* social adjustment instrument for children and adolescents, *SNAP-IV* Swanson, Nolan, and Pelham

^a^14 students with missing value in gender variable were found

**Fig. 1:**
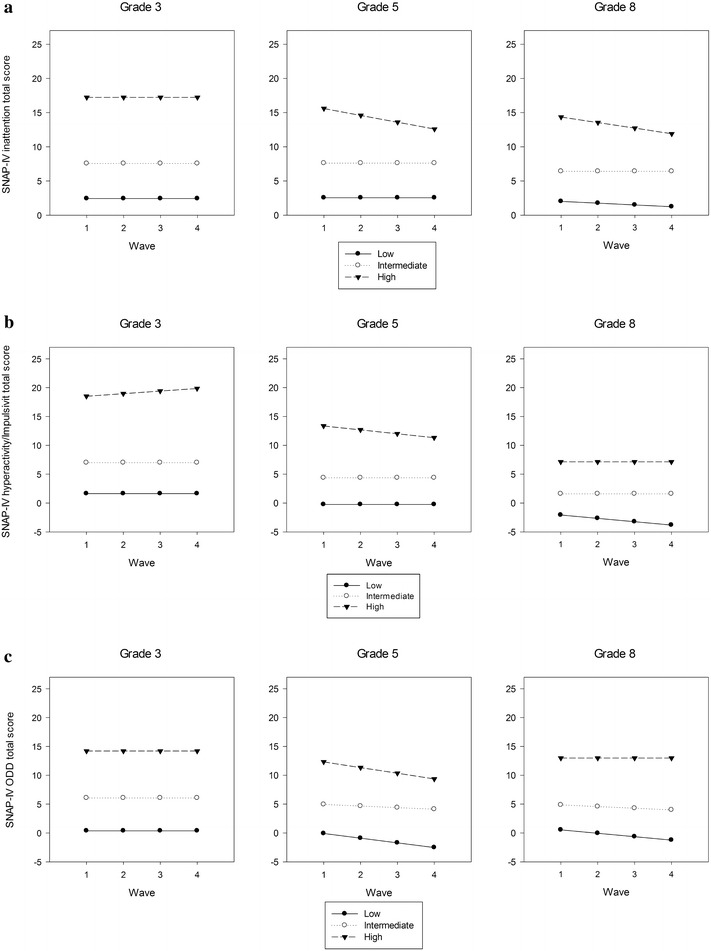
Group based trajectory modelling analyses for each grade using the respective subscale of the SNAP-IV. **a** Inattention domain. **b** Hyperactivity–impulsivity domain. **c** Oppositional-defiance domain

Group-based trajectory modeling analyses according to four waves of the SNAP-IV subscales identified three trajectories in each symptom domain, classifying them as Low, Intermediate and High symptomatic severity groups based on their persistence of symptoms over time (Fig. 1a–c). The proportions of the three symptomatic level groups are presented as follows: Low (29.0%; mean ± SD 2.44 ± 1.26), Intermediate (58.5%; mean ± SD 6.95 ± 1.61), and High (12.5%; mean ± SD 14.61 ± 2.97) in the IA domain; Low (40.6%; mean ± SD 1.61 ± 1.16), Intermediate (52.5%; mean ± SD 5.82 ± 1.80), and High (6.9%; mean ± SD 13.86 ± 3.02) in the HI domain; Low (34.1%; mean ± SD 1.20 ± 0.84), Intermediate (57.4%; mean ± SD 5.11 ± 1.63), and High (8.5%; mean ± SD 12.66 ± 2.79) in the OD domain, respectively.

Figure 1 illustrate the group-based trajectory modelling analyses for each grade using the IA (Fig. 1a), HI (Fig. 1b), and OD (Fig. 1c) subscales of the SNAP-IV.

Subgroup analyzes for each grade showed separated three levels of trajectories (Low, Intermediate and High) in each symptom domain (Fig. 1a, IA; b, HI; c, OD). For these different severity levels, two patterns (i.e., shapes) were found; the first was a quadratic or linear model in which ADHD symptoms decreased slowly over time. The other was an intercept-only model in which ADHD symptoms remained steady over time. Trajectory pattern differed slightly between grades. For example, trends with quadratic decreasing patterns were noted in High symptomatic severity trajectories of the IA and HI domains in grade 3; whereas, a linear decreasing pattern was found in High trajectories of the IA and HI domains in grade 8, but not those in grade 5. In the OD domain, High trajectories in grades 3, 5, and 8 were steadily flat, quadratic decreasing, and linear decreasing, respectively.

Table 2 shows demographics and baseline behavioral and emotional problems, perceived family function, and social and school adjustments at the first wave across the ADHD symptom domains, as well as separated by symptom severity. Table 3 presents a comparison between three severity groups (Intermediate vs. Low, High vs. Low) using stepwise multinomial logistic regression to identify factors that differentiated the trajectories. Generally speaking, we found that male gender, more externalizing problems, fewer prosocial behaviors, lower school function, more behavioral problems at home, and less perceived family support could differentiate the High trajectories from the Low trajectories in each symptom domain. Among these variables, poor school function (odds ratio OR = 1.23, 95% confidence interval CI 1.16–1.30 in the IA domain; OR = 1.55, 95% CI 1.42–1.68 in the HI domain; OR = 1.32, 95% CI 1.22–1.42 in the OD domain) and less prosocial behavior (OR = 0.87, 95% CI 0.78–0.97 in the IA domain; OR = 0.68, 95% CI 0.58–0.81 in the HI domain; OR = 0.67, 95% CI 0.59–0.77 in the OD domain) were most consistent across symptom domains. We found that similar variables in the model with different degrees of impact could differentiate the Intermediate trajectory from the Low trajectory. Besides, male students, when compared to female, could differentiate High and Low trajectories in the IA and HI domains but not in the OD domain. More emotional symptoms and conduct problems of the students were found to differentiate High from Low trajectories in the IA and OD domains, but this was not true of students in the HI domain. Lower school grade level differentiated High from Low and Intermediate from Low trajectories in both HI and OD domains but not in the IA domain.

**Table 2:** Behavioral and emotional problems, perceived family function and adjustments in different severity trajectories among three symptom-domain groups

Variables^b^	IA group			HI group			OD group		
	Low (N = 372)	Intermittent (N = 749)	High (N = 160)	Low (N = 520)	Intermittent (N = 672)	High (N = 89)	Low (N = 437)	Intermittent (N = 735)	High (N = 109)
Gender, n (%)^a^									
Male	140 (37.74)	396 (53.73)	111 (69.81)	229 (44.21)	351 (53.02)	67 (77.01)	203 (46.67)	383 (52.83)	61 (57.01)
Female	231 (62.26)	341 (46.27)	48 (30.19)	289 (55.79)	311 (46.98)	20 (22.99)	232 (53.33)	342 (47.17)	46 (42.99)
Grade									
Grade 3	51 (13.71)	177 (23.63)	36 (22.50)	53 (10.19)	171 (25.45)	40 (44.94)	60 (13.73)	171 (23.27)	33 (30.28)
Grade 5	81 (21.77)	159 (21.23)	43 (26.88)	99 (19.04)	155 (23.07)	29 (32.58)	87 (19.91)	162 (22.04)	34 (31.19)
Grade 8	240 (64.52)	413 (55.14)	81 (50.62)	368 (70.77)	346 (51.49)	20 (22.47)	290 (66.36)	402 (54.69)	42 (38.53)
SDQ, mean (SD)									
Conduct problems	1.71 (0.87)	1.98 (1.08)	3.00 (1.66)	1.75 (0.88)	2.12 (1.19)	3.28 (1.81)	1.77 (0.91)	1.99 (1.13)	3.41 (1.56)
Hyperactivity	3.43 (1.03)	3.72 (1.43)	4.35 (1.47)	3.37 (1.1)	3.82 (1.42)	5.07 (1.34)	3.52 (1.25)	3.74 (1.38)	4.34 (1.39)
Emotional symptoms	1.37 (1.59)	2.07 (1.86)	3.21 (2.21)	1.59 (1.66)	2.21 (1.99)	3.14 (2.28)	1.50 (1.66)	2.13 (1.87)	3.33 (2.37)
Peer problems	4.45 (1.23)	4.4 (1.31)	4.54 (1.53)	4.49 (1.27)	4.32 (1.34)	4.80 (1.42)	4.56 (1.30)	4.33 (1.29)	4.50 (1.51)
Prosocial	8.14 (1.89)	7.31 (2.02)	6.41 (2.11)	7.85 (1.98)	7.22 (2.08)	6.54 (2.02)	8.18 (1.89)	7.16 (2.00)	6.13 (2.07)
Family APGAR, mean (SD)	7.85 (2.6)	6.83 (2.99)	6.08 (3.28)	7.45 (2.67)	6.74 (3.12)	6.81 (3.20)	7.52 (2.76)	6.88 (3.03)	6.08 (3.11)
SAICA, mean (SD)									
School function	11.89 (2.00)	13.94 (3.48)	18.88 (5.10)	12.41 (2.31)	14.59 (4.06)	19.30 (5.85)	12.47 (2.58)	14.19 (3.80)	18.6 (5.79)
Home behaviors	20.74 (5.67)	21.95 (6.60)	25.41 (7.45)	21.2 (5.72)	22.56 (6.89)	23.80 (8.78)	20.34 (5.50)	22.35 (6.47)	27.17 (8.33)

*SD* standard deviation, *IA* inattention, *HI* hyperactivity–impulsivity, *OD* oppositional-defiance, *SDQ* Strengths and Difficulties Questionnaire, *Family APGAR* family adaptation, partnership, growth, affection, and resolve, *SAICA* social adjustment instrument for children and adolescents

^a^14 students with missing value in gender variable were found

^b^Only first wave data of were used in the analysis

**Table 3:** Stepwise multinomial logistic regression of trajectory groups on demographics, baseline behavioral and emotional problems, perceived family function and social adjustments

Variables^b^	IA group		HI group		OD group	
	OR (95% CI)		OR (95% CI)		OR (95% CI)	
	Intermediate vs. low	High vs. low	Intermediate vs. low	High vs. low	Intermediate vs. low	High vs. low
Gender						
Male vs. female	0.55 (0.41–0.75)***	1.93 (1.21–3.08)***	1.25 (0.94–1.66)	4.04 (1.95–8.38)***	–^a^	–^a^
Grade (Ref = grade 3)						
Grade 5	1.52 (0.94–2.46)	1.16 (0.62–2.16)	0.54 (0.34–0.85)***	0.32 (0.14–0.70)***	0.80 (0.51–1.25)	0.67 (0.32–1.43)
Grade 8	2.73 (1.79–4.17)***	0.80 (0.46–1.41)	0.18 (0.12–0.27)***	0.02 (0.01–0.05)***	0.36 (0.25–0.54)***	0.13 (0.06–0.26)***
SDQ						
Conduct problems	0.96 (0.81–1.13)	1.31 (1.09–1.58)**	–^a^	–^a^	1.07 (0.92–1.25)	1.78 (1.41–2.23)***
Hyperactivity	0.88 (0.77–0.99)*	1.15 (0.99–1.35)	1.36 (1.20–1.53)***	2.58 (2.03–3.26)***	–^a^	–^a^
Emotional symptoms	0.87 (0.79–0.97)*	1.10 (0.98–1.23)	–^a^	–^a^	1.10 (1.01–1.20)***	1.20 (1.04–1.39)*
Peer problems	–^a^	–^a^	0.86 (0.76–0.96)***	1.22 (0.96–1.55)	–^a^	–^a^
Prosocial	1.20 (1.11–1.31)***	0.87 (0.78–0.97)*	0.89 (0.83–0.96)**	0.68 (0.58–0.81)***	0.78 (0.72–0.84)***	0.67 (0.59–0.77)***
Family APGAR	1.09 (1.03–1.15)***	0.93 (0.86–1.00)*	–^a^	–^a^	–^a^	–^a^
SAICA						
School function	0.72 (0.67–0.79)***	1.23 (1.16–1.30)***	1.29 (1.22–1.36)***	1.55 (1.42–1.68)***	1.18 (1.11–1.24)***	1.32 (1.22–1.42)***
Home behaviors	–^a^	–^a^	–^a^	–^a^	1.04 (1.01–1.07)**	1.13 (1.08–1.18)***

*OR* odds ratio, CI confidence interval, *IA* inattention, *HI* hyperactivity–impulsivity, *OD* oppositional-defiance, *SDQ* Strengths and Difficulties Questionnaire, *Family APGAR* family adaptation, partnership, growth, affection, and resolve, *SAICA* social adjustment instrument for children and adolescents

^a^ Non-significant variable

^b^Only first wave data of were used in the analysis

* p < 0.05, ** p < 0.01, *** p < 0.001

## Discussion

In order to explore different trajectories of IA, HI and OD symptoms and their associated factors among children and adolescents, this community-based study identified three trajectories (Low, Intermediate, and High) of three symptom domains (IA, HI and OD) with various correlates of demographics, emotional and behavioral symptoms, family function, and school and social adjustment. Poor school function and less prosocial behaviors were the most consistent associated factors across the three symptom models that differentiated High to Low trajectories in different grades and could be used as a marker to identify patients at risk of ADHD in a community setting.

The proportion of participants classified as a High trajectory in subgroups IA, HI, and OD were 12.5, 6.9, 8.5%, respectively. The majority of participants were in the Low and Intermediate symptom trajectories. The proportion of participants in High symptom severity trajectories (6.9–12.5%) is similar to our previous findings of ADHD prevalence (7.5%) by semi-structured psychiatric interview of randomly selected school samples in Taiwan [2]. The students in the High severity group had more severe behavioral problems and perceived fewer family supports assessed by the SAICA and family APGAR, similar to the impression of children with a formal diagnosis of ADHD [34]. On the other hand, children and adolescents in the Low and Intermediate trajectory groups could be considered as their ‘normally developing’ counterparts, demonstrating slight to moderate ADHD traits. Collectively, we could postulate that the substantial proportion of students in the High symptom trajectories might represent community samples of ADHD [2].

Students in High symptom trajectories were found to be associated with more severe externalizing behaviors and poorer school and home adjustment comparing to the Low or Intermediate symptom subgroups. This is consistent with a previous community-based study showing more severe externalizing and internalizing symptoms and a lower quality of life in high ADHD symptom trajectories [14]. Having psychiatric comorbidities such as ODD, conduct, bipolar, and anxiety disorders at baseline were all significant predictors of a persistent course of ADHD symptoms [7]. Low prosocial behavior at baseline and high SAICA scores on school function could differentiate the course of IA, HI, and OD in the following year and could be considered useful tools for clinical evaluation when screening for ADHD and ODD. Our findings also align with previous studies demonstrating that the hyperactivity–inattentive subscale of SDQ shows good agreement with the diagnostic criteria for ADHD [39, 40]. Further, they suggest that these factors are predictive of ADHD symptom severity after approximately 1 year. An earlier study examining empathy and prosocial behavior in children with disruptive behavior disorder and ADHD found significantly less empathic and prosocial behavior in children with disruptive behavior disorder, irrespective of the co-occurrence of ADHD; these differences remained after controlling for ADHD symptoms [41]. Our finding that low prosocial behavior was not only associated with the High trajectory in the OD domain, but also with the High trajectory in the IA and HI domains implies that child’s oppositional and ADHD behaviors should be closely monitored as atypical prosocial behaviors develop.

Poor perceived family support was associated with the High trajectory in the IA domain in the current study. Previous studies showed that poor family function increased aggression of ADHD children according to parental reports [42], and family socioeconomic status at baseline was significantly associated with initial and later ADHD severity and impairment [43]. Thus, we need to identify at-risk children as early as possible to provide personalized intervention to offset the possible aggression and impairment in later development stages. Our findings also indicated that children’s poor functioning at school and home setting—especially at school—were associated with High trajectories among all three symptom domains and across each grade. Poor school function could be considered as a proxy of functional impairment and ADHD-related symptom trajectories. Hence, children with poor functioning at school should be prioritized for intervention whether they have been diagnosed with ADHD or not.

Regarding the Intermediate and Low groups of the three symptom domains, our results showed globally flat trajectories of symptom severity. Generally suggesting a stable course and severity over time. However, not all High trajectories declined over time. Our results above contradict to the findings of previous studies of clinical patients with ADHD, which showed a persistent reduction in HI symptoms [44, 45] but a relatively constant severity in the IA domain [44–46]. These discrepancies will be explained in the following context. First, this study had a shorter follow-up duration (1 year); whereas, a previous similar study had a 4-year follow-up period [44]. Second, our sample consisted of participants across three developmental periods (i.e., childhood, pre-adolescence, and early adolescence). The trends in the three developmental periods had somewhat distinct patterns, but these differences were neutralized in the final trajectories after combining all grades together. Furthermore, the distinct patterns between IA, HI, and OD also differed by different grades, which might indicate that the developmental course of ADHD symptomatology is not straightforward and should not be analyzed globally within one group. Our finding also corresponds to previous trajectory studies of community samples. We identified three as the optimal number of trajectories among these groups as we expected [14]. This helps us learn more about their symptom course and trajectory over time and might lead to earlier diagnosis if the child showed high ADHD symptom severity at the time of evaluation. Despite the short follow-up duration in our study, the finding that symptom severity trajectories differed across developmental ages suggests that children with low or moderate ADHD symptom levels during young school-age, pre-adolescence, and young adolescent periods might not be at risk for subsequent development of serious ADHD symptoms in the future, indirectly support the viewpoint that ADHD is a neurodevelopmental disorder with onset in early childhood.

### Strengths and limitations

Our study had several strengths. First, the large sample size decreased the possibility of type II errors. Second, the longitudinal study design made it possible to observe trends in different trajectories and compare baseline functions and problems at home and school in order to differentiate trajectories. Third, our behavioral measures were rated by both students and their parents. Multiple informants may have provided more diverse and ecologically valid evaluations of participants’ behaviors and functions.

This study was not without limitations. First, the total follow-up duration was approximately 1 year, which prevents us from observing clearer trajectory patterns that were achieved in studies with longer follow-up durations. One year is a rather short time interval to understand trajectory patterns for illness. However, this did not preclude us from differentiating three trajectories across three symptom domains. Second, we used first-wave evaluation scores (i.e., from the SDQ, family APGAR, and SAICA) to separate trajectories. Still, it is unclear whether scores were predictive of severity trajectories or whether impairment was caused by differing ADHD-symptom severity. Therefore, further investigation is needed to determine whether a causal relationship exists. Third, the measurements were made according to the student’s and parent’s reports of several questionnaires rather than teacher’s form. The absence of teacher’s rating may influence the evaluation of adjustment and symptom severity in a school setting. Fourth, considering that we collected data from urban samples in Northern Taiwan, results may not be generalizable to other areas in Taiwan. Lastly, a lack of formal clinical ADHD diagnosis and no records of psychostimulant use but assessment of ADHD symptoms and OD symptoms as evaluated by the SNAP-IV have impeded us from the direct comparisons with earlier studies that examined clinical samples. However, our previous clinical studies have clearly demonstrated that ADHD diagnosis is associated with, more emotional/behavioral problems, less family support and more functional impairment in school and at home [47–49]. Hence, the factors associated with High vs. Low trajectories are typical of those associated with the diagnosis of ADHD and made these high trajectory samples more relevant to clinical ADHD samples. This could better characterize the ‘real-world’ problems faced by students in the community, where ADHD is underdiagnosed and less treated but caused a huge burden on the patients and their family and also impairment of their daily function.

## Conclusions

Three different trajectories (Low, Moderate, and High) for the IA, HI, and OD symptom domains were identified in a community-based sample. Two trajectory patterns, a quadratic or linear decreasing model, and an intercept-only model were noted and High trajectory in the three domains showed all linear decreasing patterns in grade 8. About 6.9–12.5% children were classified in the High trajectories of ADHD symptoms, which might be the approximate prevalence of ADHD in Taiwan. The High trajectory can be differentiated from others by the following factors: male gender, more externalizing problems, less prosocial behaviors, more severe school dysfunctions, more severe home behavioral problems, and less perceived family support. Among these predictors, poor school function and less prosocial behavior had the most robust influence on different levels of ADHD symptomatology. Our findings could help to develop the specific measures for managing high ADHD symptoms over time in a school setting. These findings extend the literature on ADHD trajectories and may inform future research.

## Additional file

**Additional file 1: Table S1.** Model fit for trajectories analyses.

## Abbreviations

ADHD: attention-deficit/hyperactivity disorder
IA: inattention
HI: hyperactivity–impulsivity
OD: oppositional-defiance
SDQ: Strengths and Difficulties Questionnaire
Family APGAR: family adaptation, partnership, growth, affection, and resolve
SAICA: social adjustment instrument for children and adolescents
SNAP-IV: Swanson, Nolan, and Pelham Version IV Scale
SD: standard deviation
OD: odds ratio
CI: confidence interval
ODD: oppositional defiant disorder
BIC: Bayesian information criterion
