# Supplementary material for: One-year trajectory analysis for ADHD symptoms and its associated factors in community-based children and adolescents in Taiwan
Source: Child Adolesc Psychiatry Ment Health. 2017 Jun 1;11:28. doi: 10.1186/s13034-017-0165-4 (PMC5452532; doi:10.1186/s13034-017-0165-4)
Supplement: Supplementary file 1 — Additional file 1: Table S1. Model fit for trajectories analyses. [file 13034_2017_165_MOESM1_ESM.docx]

**Additional file 1: Table S1. Model fit for trajectories analyses.**

|  | IA group | | HI group | | OD group | |
| --- | --- | --- | --- | --- | --- | --- |
| Number of trajectories | BIC (n = 5124) | BIC (n = 1281) | BIC (n = 5124) | BIC (n = 1281) | BIC (n = 5124) | BIC (n = 1281) |
| 6 | -12735.6 | -12714.8 | -10969.3 | -10948.5 | -11762.7 | -11741.9 |
| 5 | -12793.6 | -12776.3 | -11049.1 | -11031.8 | -11812.5 | -11795.2 |
| 4 | -12839.7 | -12825.9 | -11085.3 | -11071.4 | -11861.1 | -11847.3 |
| 3 (final model) | -13077.4 | -13067.0 | -11371.3 | -11360.9 | -12047.2 | -12036.8 |
| 2 | -13593.4 | -13586.5 | -11787.1 | -11780.2 | -12579.3 | -12572.3 |
| 1 | -14609.1 | -14605.6 | -12687.1 | -12683.7 | -13380.1 | -13376.7 |

BIC Bayesian information criterion, IA inattention, HI hyperactivity-impulsivity, OD oppositional-defiance
